# Crohn’s disease exacerbated by IL-17 inhibitors in patients with psoriasis: a case report

**DOI:** 10.1186/s12876-020-01474-x

**Published:** 2020-10-15

**Authors:** Jingyi Ju, Yuanyuan Dai, Jiaolan Yang, Changqin Liu, Li Fan, Lijin Feng, Binghui Zhao, Meiying Zeng, Zhanju Liu, Xiaomin Sun

**Affiliations:** 1 Department of Gastroenterology, Shanghai Tenth People’s Hospital of Tongji University, No. 301 Yanchang Road, Shanghai, 200072 China; 2 Department of Pathology, Shanghai Tenth People’s Hospital, Tongji University, Shanghai, China; 3 Department of Radiology, Shanghai Tenth People’s Hospital, Tongji University, Shanghai, China; 4 Department of Gastroenterology, Shanghai Tenth People’s Hospital Chongming Branch, No. 66 Xiangyang East Road, Chongming, 202157 China

**Keywords:** Psoriasis, Inflammatory bowel disease, Crohn’s disease, IL-17 inhibitor, IL-23 inhibitor

## Abstract

**Background:**

Previous studied revealed that psoriasis and Inflammatory bowel disease (IBD) have highly overlapping epidemiological characteristics, genetic susceptibility loci, disease risk factors, immune mechanisms, and comorbidities. More and more biologics have been used to treat psoriasis and IBD. Interleukin (IL)-17 inhibitors played an important role in the treatment of psoriasis, but induced and aggravated inflammatory bowel disease in some patients. IL-23 inhibitors have shown to be effective to both psoriasis and CD.

**Case presentation:**

Forty-one year old Chinese male patient who came to the hospital for psoriasis, developed severe gastrointestinal symptoms after using an IL-17 inhibitor, and was diagnosed with Crohn’s disease (CD). The patient eventually used an IL-23 inhibitor to relieve both psoriasis and CD.

**Conclusion:**

IBD patients and psoriasis patients have increased probability of suffering from the other disease. The case that patients had suffered from psoriasis and CD before the use of IL-17 inhibitor is quite rare. This case suggests that physicians need to be careful when treating patients with psoriasis and CD with biologics, and it is necessary to evaluate the gastrointestinal tract.

## Background

Psoriasis is a chronic, recurrent, inflammatory skin disease caused by the combination of immune, environmental, and psychological factors in a genetic background. Inflammatory bowel disease (IBD) that includes Crohn’s disease (CD) and ulcerative colitis (UC) is a non-specific intestinal inflammatory disease with unknown etiology and pathogenesis, which may be related to genetic susceptibility, intestinal flora, intestinal mucosal barrier dysfunction, environment, diet, mental and other factors. Both psoriasis and CD show impaired physical barriers in skin and intestine respectively. Previous studies revealed that psoriasis and IBD have highly overlapping epidemiological characteristics, genetic susceptibility loci, disease risk factors, immune mechanisms, and comorbidities. IBD patients and psoriasis patients have increased probability of suffering from the other disease [1, 2]. Although some clinical cases about the use of interleukin (IL)-17 blockers inducing CD have been reported, the patient had suffered from psoriasis and CD before the use of IL-17 inhibitor is quite rare. The case introduces a patient with psoriasis and CD whose CD exacerbated by IL-17 inhibitors.

## Case presentation

We report a 41-year-old Chinese male patient who went to the Department of Gastroenterology, Shanghai Tenth People’s Hospital for treatment. He suffered from skin lesions and diarrhea for more than 3 years, perianal abscess and bloody stool for 2 years, and exacerbation of abdominal pain for 1 year. The patient had a 25-year history of smoking and didn’t have a family history of CD, however, his grandfather, father, and cousin also had psoriasis. Tracing back the medical history, the patient developed erythema and desquamation on the trunk and limbs from the summer of 2016. He was diagnosed as psoriasis at the Department of Dermatology of Shanghai Huashan Hospital. Using oral silver-removing granules (traditional Chinese medicine) and topical calcipotriol cream did not improve the skin lesions efficiently. The symptoms of patient included the diarrhea as well as increased frequency of defecation. Given no obvious abdominal pain, pus and blood, he did not see a doctor. By the beginning of 2017, the patient felt perianal discomfort, and there was hematochezia and the yellow sticky discharge, which was diagnosed as perianal abscess at Anorectal Surgery, Shanghai Shuguang Hospital, where the patient was given symptomatic treatment. After that, the patient’s perianal abscess improved, but there was still blood in the stool.

**Fig. 1:**
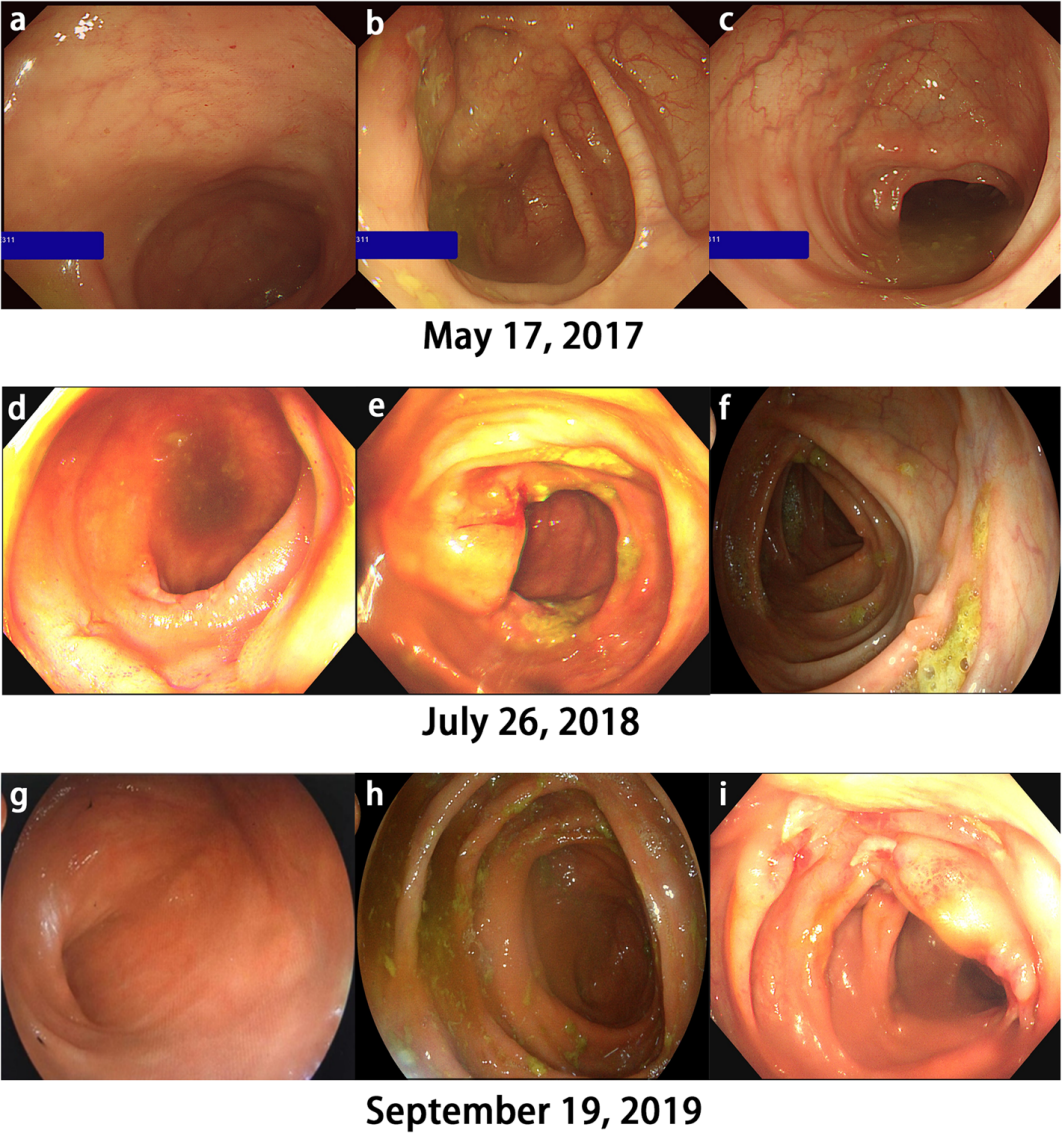
Colonoscopy showed from **a** to **c**, scattered aphthous ulcers in the terminal ileum, ileocecal region and descending colon before the use of IL-17 inhibitors and from **d** to **i**, the progression from severe Crohn’s colitis with deep punch out ulcers after the use of IL-17 inhibitors to healed mucosa in endoscopic remission following anti-TNF and IL-23 inhibitor therapy

**Fig. 2:**
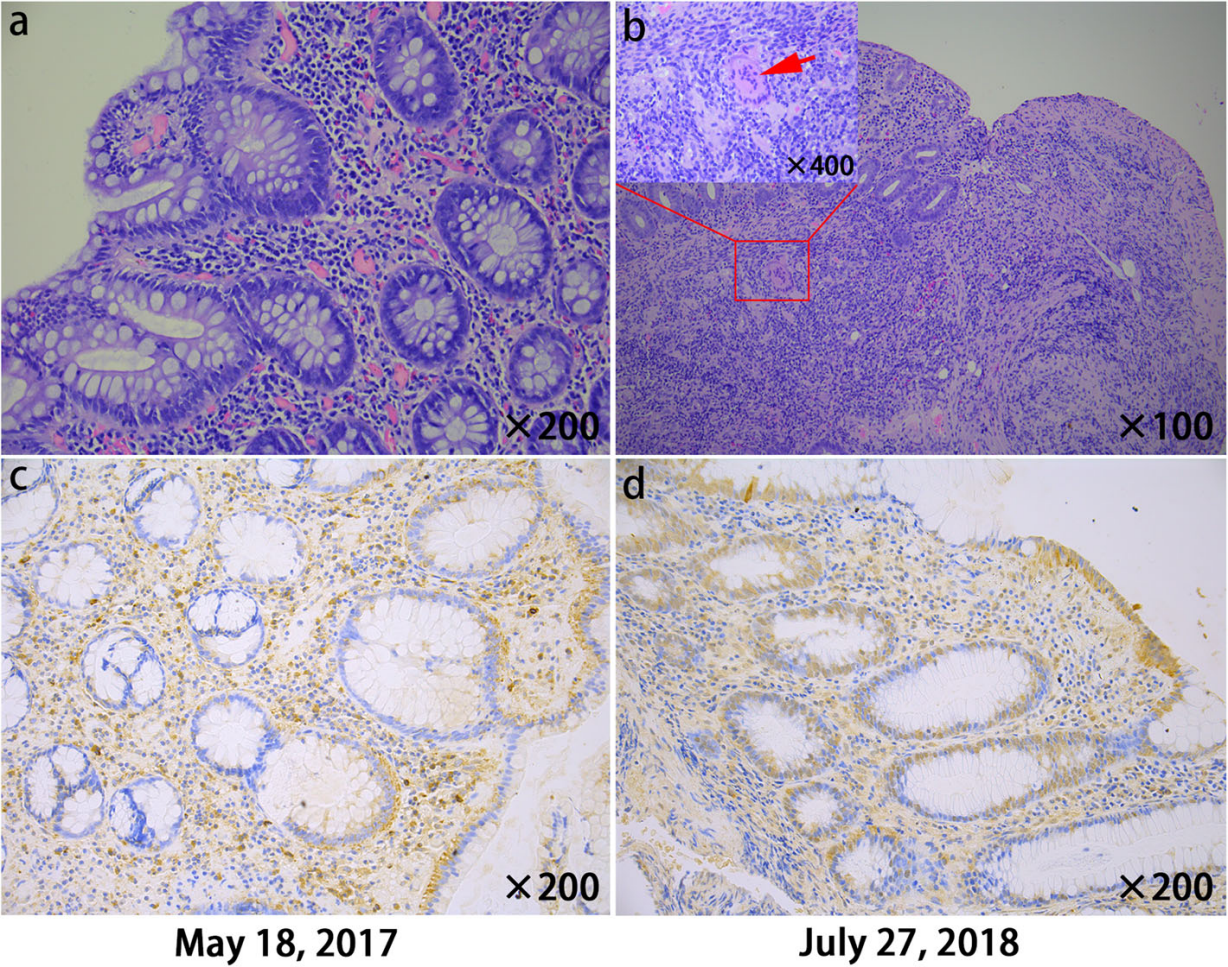
Pathology **a** demonstrated the chronic active inflammation of the mucosa in the ileocecal junction. **b** showed submucosal langerhans giant cells in the colon, considered as granulomatous inflammation. Crypts disappeared in part of the mucous membrane area, but the fissure ulcer was not obvious. Immunohistochemical staining in image **c** showed moderate intensity staining of inflammatory cells in the lamina propria of the mucosa, mainly plasma cells, neutrophils and monocytes, and the glandular epithelium of the crypts was weakly expressed. **d** showed moderate intensity staining of inflammatory cells in the lamina propria and the glandular epithelium of the crypts was widely expressed

He was performed medical exams in May 17, 2017, and the colonoscopy showed scattered aphthous ulcers in the terminal ileum (Fig. 1a), ileocecal region (Fig. 1b) and descending colon (Fig. 1c) before the use of IL-17 inhibitors. A pathology of his ileocecal junction indicated chronic active inflammation of the mucosa (Fig. 2a).

In June 2017, the patient’s skin lesions worsened, and he returned to Shanghai Huashan Hospital to use IL-17 inhibitor secukinumab, 0.3 g per week for 4 weeks, and then 0.3 g per month, a total of 8 months.

In July 2017, 1 month after the application of secukinumab, the skin lesions significantly improved, but mucopurulent bloody stool appeared, about 5 times per day, with obvious pain in mid-lower abdomen, which eased slightly after defecation.

From August 2017 to March 2018, the patient used the IL-17 inhibitor. During this period, the patient used 0.045 g of ustekinumab (IL-12 / 23 inhibitor) once, and the skin lesions did not improve.

From April to June 2018, the patient received ixekizumab (IL-17A antagonist), 0.08 g per month.

In July 2018, the patient’s skin lesions healed, but the intestinal symptoms worsened, so he went to the Department of Gastroenterology, Shanghai Tenth People’s Hospital, where laboratory investigations were significant for an elevated Creactive protein (CRP) of 0.0765 g/L and the reduced Hemoglobin (HB) of 120 g/L.

After the use of IL-17 inhibitors, the colonoscopy showed longitudinal ulcer and multi-segment ulcers in the distal ileum and colorectum, congestion and edema in the surrounding mucosa, and paving stone-like changes (Fig. 1d, e, f) at locations similar to Fig. 1a, b, c, respectively.

**Fig. 3:**
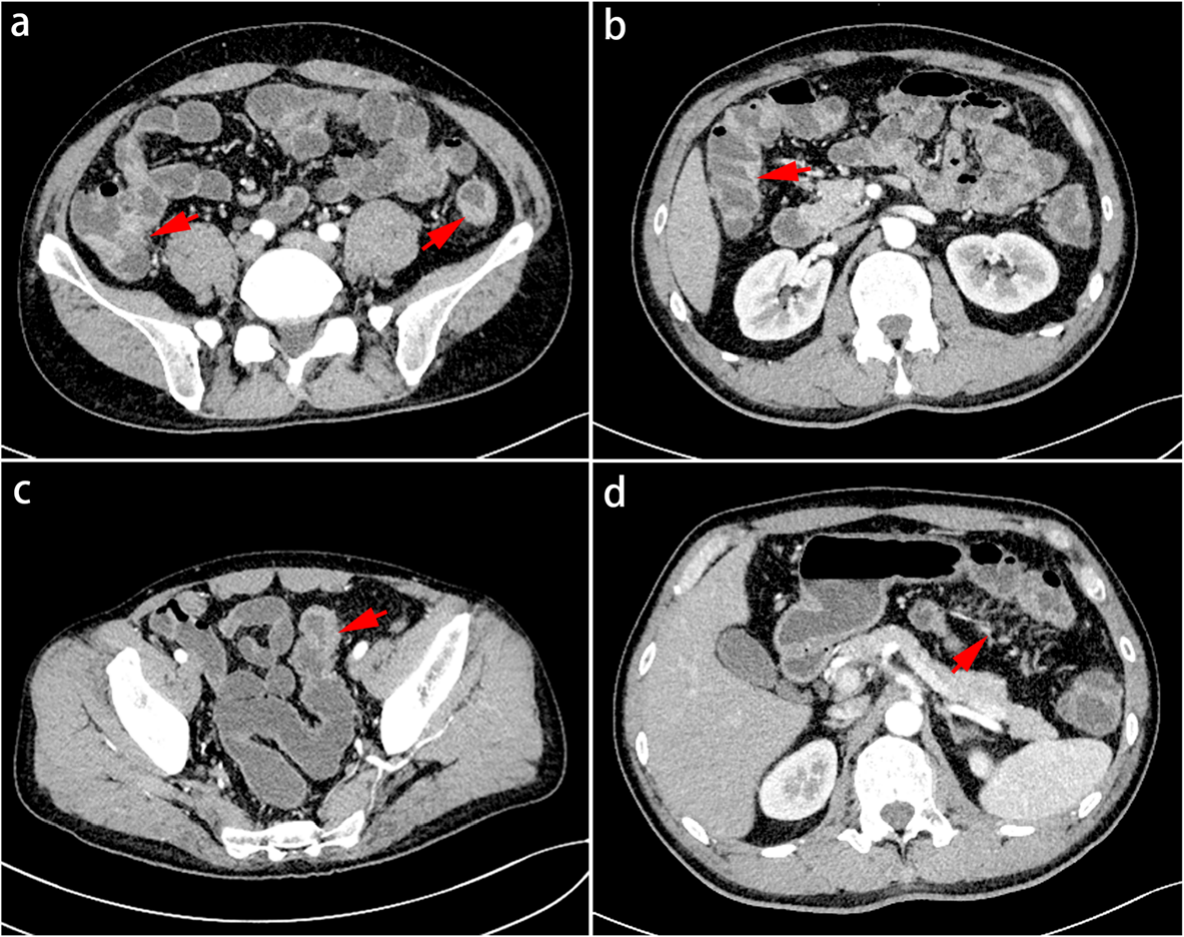
The CT of intestine demonstrated mesentery-side blood vessels around the intestinal loop proliferated, dilated, and twisted. Straight vessels formed comb-like shape. Thickened intestinal wall and lumen mild stenosis were observed from ileocecum to ascending colon, sigmoid colon and the descending colon in turn

The intestine CT demonstrated that the jejunum, ileum, ileocecal junction (Fig. 3a, left arrow), ascending colon (Fig. 3b), transverse colon, descending colon (Fig. 3a, right arrow), and sigmoid colon (Fig. 3c) have a long range of intestinal wall thickening to varying degrees, poor filling and expansion, and mild intestinal lumen narrowing. Correspondingly, mesentery-side vessels around the intestinal loop proliferated, dilated, and twisted. Small straight vessels expanded to form the comb-like shape (Fig. 3d). There were multiple small lymph nodes around some mesentery vessels. Therefore, he was diagnosed with Crohn’s disease in active phase.

The pathology of colon revealed the hyperemia and erosion of the mucosa, infiltration of moderate lymphocytes, plasma cells and a small amount of eosinophils and neutrophils in the lamina propria, hyperplasia of lymphoid tissue in the submucosa, suspicious granulomas and suspicious fissure ulcer (Fig. 2b).

Immunohistochemical staining showed moderate intensity staining of inflammatory cells in the lamina propria, and the glandular epithelium of the crypts was weakly expressed before the use of IL-17 inhibitors (Fig. 2c). After the use of IL-17 inhibitors the immunohistochemical staining also showed moderate intensity staining of inflammatory cells in the lamina propria, but the glandular epithelium of the crypts was widely expressed.

The physical exams showed extensive dark brown patches of skin. Scattered patches of erythema and desquamation was on the limbs and trunk. Perianal swelling and a little purulent discharge could be seen. He had lost 10 kg of weight since his illness. From July 25, 2018, the patient took an oral immunosuppressant: azathioprine 0.05 g daily, and felt unwell. From September 10, 2018, he reduced it to 0.025 g daily, and stopped using it on October 30, 2018.

In 2018-7-31, 8–13, 9–10, 10–30, 12–14, and 2019-02-15, the patient was treated with Infliximab 0.4 g 6 times totally.

After the first treatment with infliximab, the purulent stool and abdominal pain disappeared, and there was no perianal exudation.

After the four times use of infliximab, the patients gradually appeared joint pain and increased skin lesions. It was suggested to continue to use infliximab after consultation with dermatology department, but the symptoms of joints and skin lesions were not relieved. The patient was worried. Infliximab was stopped after 6 times of use.

From April 3, 2019, the patient was treated with Tremfya (guselkumab, IL-23 inhibitor) 0.1 g per piece at week 0,4, and then every 8 weeks, maintained until December 9, 2019 for a total of 6 times. After the second treatment with guselkumab, the rash disappeared and the joint pain disappeared. During this period, the patient had no intestinal symptom, so he was satisfied with his quality of life.

In September 2019, the patient was re-examined. The serum IL-17A was 6.73 pg/ml. The colonoscopy showed that the previous longitudinal ulcers healed and polyp-like hyperplasia was seen, which was considered as Crohn’s disease in remission following anti-TNF and IL-23 inhibitor therapy (Fig. 1g, h, i).

## Discussion and conclusions

Compared with the patient who used IL-17 inhibitor, and was eventually diagnosed with CD, the male patient in this case presented with psoriasis, and more severe gastrointestinal symptoms. Reviewing his medical history, his grandfather, father, and cousin also had psoriasis. However, the patient does not have a family history of CD. The patient has a 25-year history of smoking and has not stopped smoking, which is a possible risk factor for CD [3].

We found that in his first time to see a doctor for psoriasis, he also had mild gastrointestinal symptoms including deformed stools and increased frequency of defecation.

Perianal abscess is one of the characteristic complications of Crohn’s disease. According to the patient’s colonoscopy, pathology, and perianal abscess in 2017, we highly suspect that the patient had potential Crohn’s disease before using the IL-17 inhibitor, although CD was not confirmed at the time. Therefore, the patient in this case was considered CD with plaque psoriasis.

Psoriasis and CD have some similarities in treatment. Common drugs include glucocorticoids, immunosuppressants and biologics. Biologics are currently new types of drugs with targeted therapies, while patients are afraid of these new drugs [4].

The patient in this case had used multiple biological agents. Before the diagnosis of Crohn’s disease, IL-17A inhibitors and IL-12 / 23 inhibitors were used, mainly for the treatment of psoriasis. After the diagnosis of Crohn’s disease, Infliximab and IL-23 inhibitors were used.

Previous research revealed that secukinumab and ixekizumab are effective for psoriasis and psoriasis arthritis, but may induce or exacerbate IBD [5]. In the treatment of psoriasis, compared with ustekinumab, ixekizumab showed higher efficacy and safety for 52 weeks [6].IL-17A plays an important role in the pathogenesis of inflammation of psoriasis. Data from clinical trials have shown that ixekizumab can effectively treat patients with moderate and severe psoriasis by inhibiting IL-17A and reducing plaque formation [7, 8]. Th17 cells release IL-17A, which increases in the serum, intestinal mucosa, and stool of active IBD patients [9]. A large amount of Th17, IFN-y, IL-6, IL-17, IL-22 and IL-23 were also found in skin lesions of patients with psoriasis [10]. However, the efficacy of IL-17 inhibitors as a therapeutic target for IBD is bad. There is no reliable evidence for treating patients with moderate and severe active CD with IL-17 inhibitors [11]. IL-17 has a protective effect on intestinal inflammation, inducing protective intestinal epithelial gene expression, and increases the mucosal defense function against intestinal microorganisms [12]. Maxwell et al. used the mouse model of IBD. Then, he found that the inhibition of IL-17 weakened the intestinal epithelial barrier function and aggravated the inflammation. This phenomenon was related to the inhibition of IL-17A the increase of intestinal epithelial permeability, the decrease of the expression of antimicrobial peptide and the decrease of neutrophil aggregation [13]. In a clinical trial of secukinumab in 59 patients with severe CD, IL-17inhibitors were not only ineffective, but also associated with adverse events and CD deterioration [14]. A recent review by Hohenberger et al. also talked about the relationship between IL-17 inhibition and exacerbation of colitis. Comparing experimental data in mice with clinical trial data, they found that inhibiting IL-17A intestinal inflammation further worsens the disease [15]. In previous studies, IL-12 / 23 inhibitors have shown certain efficacy in psoriasis and Crohn’s disease, but in this case, IL-12 / 23 inhibitors failed to improve skin lesions, which may be related to the less use of IL-12 / 23 inhibitors only once, the serious clinical manifestations of patients and the long-term use of IL-17 inhibitors [16].

The common susceptibility gene IL23R for psoriasis and Crohn’s disease encodes IL-23. IL12B encodes the p40 protein subunit common to IL-12 and IL-23. IL-12 and IL-23 have important effects on the innate and acquired immunity. Dendritic cells and macrophages produce IL-12 and IL-23. IL-12 can stimulate the differentiation of type I innate immune cells (ILC1) and produce cytokines such as interferon gamma (IFN-γ) and anti-tumor necrosis factor (anti-TNF). IL-23can stimulate the differentiation of ILC3, γδT cells and natural killer T (NKT) cells and produce inflammatory factors such as IL17A, IL17F, IL22 [17, 18]..

The IL-12 / 23 pathway works on adaptive immunity. IL-12 can directly stimulate CD4 + helper T cells to differentiate into Th1 cells, which can produce IFN-γ. IL-23 stimulates memory CD4 + T cells through the STAT3 signaling pathway to secrete a large number of inflammatory mediators such as IL-17, IL-6, and TNF α and IFN-γ [19]. Under the comprehensive action of special environment and other cytokines such as TGF β, IL-23 promotes the expression of Th17 cells and stimulate Th17 cells to secrete IL-17 and TNF, which have proinflammatory effects [20] . The IL-12/IL-23 inhibitor, ustekinumab, can bind to P40 and inhibit the differentiation of Th1 and Th17 cells [21].

Ustekinumab has significant effect on moderate and severe psoriasis. It can also maintain intestinal symptom relief, especially in patients with moderate or severe active CD who are ineffective for INF-α [22]. Infliximab is the earliest biological agent used to treat CD. In previous studies, infliximab is effective against psoriasis, colitis and CD. Infliximab is the first biological agent for the treatment of CD [5]. The patient’s bowel symptoms improved after using IFX, but the skin lesions worsened and joint pain occurred. Therefore, infliximab is effective for Crohn’s disease in the current case, but not effective for psoriasis. In addition, Ko et al. had reported exacerbation of psoriasis due to treatment with TNF-α antagonists [23].

Guselkumab is a human monoclonal Immunoglobulin G1λ(IgG1λ) antibody that can selectively bind to p19 subunit to inhibit the interaction of IL-23 with the receptor and disrupt the Th17 cell / IL-17 pathway. IL-23 inhibitors have good safety and have significant effects on moderate to severe psoriasis [24]. In this case, guselkumab also relieved CD.

In the other case, a patient finally used IL-12/IL-23 inhibitors to alleviate psoriasis and Crohn’s disease [16]. In our case, the patient used IL-23 inhibitors with good effect. A case report by Grossberg L [25] also confirmed the dual response of psoriasis and Crohn’s disease to use of IL-23 specific blockade using guselkumab. A review by Ma C [26] summarized the research progress of IL-12/IL-23 inhibitors and IL-23 inhibitors and affirms their effectiveness and safety. Overall, IL-23 inhibitors have great potential in treating Crohn’s disease.

In summary, we introduce a 41-year-old male patient with psoriasis and CD who has aggravated CD after receiving IL-17 inhibitors for psoriasis. This patient has a 25-year history of smoking, a risk factor for Crohn’s disease. Although the patient was not diagnosed with Crohn’s disease at the first diagnosis of psoriasis, based on the patient’s clinical manifestations and subsequent examination results, we inferred that the patient was psoriasis with Crohn’s disease. This case suggests that we should pay attention to patients with psoriasis and intestinal symptoms. The gastrointestinal tract needs to be evaluated before using biologics for psoriasis. It makes sense for doctors to be careful to choose biologics for patients with psoriasis and CD.

## Data Availability

The datasets used during the current study are available from the corresponding author on reasonable request.

## Abbreviations

IBD: Inflammatory bowel disease
CD: Crohn’s disease
UC: Ulcerative colitis
IL: Interleukin
CRP: Creactive protein
HB: Hemoglobin
ILC1: Type I innate immune cells
IFN-γ: Interferon gamma
anti-TNF: Anti-tumor necrosis factor
NKT: Natural killer T cells
IgG1λ: Immunoglobulin G1λ
